# Diagnostic dilemma in a child with congenital heart disease on sildenafil

**DOI:** 10.4103/0253-7613.55203

**Published:** 2009-06

**Authors:** Syed Ahmed Zaki, Deepak Dadge, Shujaath Asif, Preeti Shanbag

**Affiliations:** Department of Pediatrics, Lokmanya Tilak Municipal Medical College & General Hospital, Sion, Mumbai 400 022, India

**Keywords:** Adverse effects, congenital heart disease, pulmonary hypertension, sildenafil

## Abstract

Sildenafil is used in infants and children mainly for the treatment of pulmonary hypertension associated with congenital heart disease. Some side-effects of sildenafil are similar to the symptoms and signs of congestive heart failure in children. We present a case of a two-month-old infant with supracardiac total anomalous venous connection and pulmonary hypertension, who presented with severe sweating and tachycardia after institution of sildenafil therapy. He improved dramatically on discontinuation of the drug.

Sildenafil is a selective phosphodiesterase inhibitor and has a potent pulmonary vasodilator effect.[1] In adults, it is used primarily in the treatment of erectile dysfunction. In children, it is used in those suffering from idiopathic pulmonary artery hypertension (PAH) or PAH associated with congenital heart disease. Some of the adverse effects of sildenafil, such as tachycardia and sweating, overlap the symptoms of congestive cardiac failure in infants and small children, thus causing a diagnostic dilemma.

## Case Report

A two-month-old male infant presented with complaints of generalized sweating more on the head, causing wetting of the pillow, for five days. There was no history of refusal of feeds, lethargy or oliguria. The infant had been admitted two weeks earlier with complaints of cough, breathlessness, and dysphonia. He had then been diagnosed, on the basis of two-dimensional echocardiography, with supracardiac total anomalous pulmonary connection (TAPVC), with severe pulmonary hypertension. The infant had been started on antifailure therapy with digoxin and furosemide. Sildenafil in a dose of 0.3 mg/kg, thrice a day, was started for the pulmonary hypertension. Following institution of the therapy, there was improvement in the infant's activity and feeding. Tachypnea and hepatomegaly also decreased. Definitive surgical management was planned and the patient was discharged.

On examination at this admission, the child was active and afebrile with a heart rate of 180/minute, respiratory rate of 40/minute, and blood pressure of 88/50 mmHg in the right upper arm. There was mild central cyanosis. Generalized sweating was present, more on the forehead. Examination of the cardiovascular system revealed a normal first heart sound, a loud second heart sound, and an ejection systolic murmur of grade 3/6 in the left second intercostal space. Respiratory and abdominal examination was normal. Considering an adverse reaction to sildenafil as a cause of symptoms, the drug was stopped. Other antifailure management was continued with the same doses. The patient showed a dramatic improvement after discontinuation of sildenafil, with a decrease in his heart rate. There was no further sweating. The patient is now awaiting corrective surgery for his cardiac lesion.

## Discussion

Pulmonary artery hypertension is defined as an increase in the mean pulmonary arterial pressure of more than 25 mmHg at rest or more than 30 mmHg during exercise, in the absence of left heart disease.[2] It can be idiopathic or secondary to congenital heart disease, with increased pulmonary blood flow. Irrespective of the cause of PAH, the end result is proliferation and remodeling of the vascular endothelium, leading to right ventricular failure. The main mechanism implicated in the pathogenesis of the disease is endothelial cell dysfunction, which leads to a reduced production of endogeneous vasodilators (prostacyclin and nitric oxide) and increased synthesis of vasoconstrictors such as endothelin-1 and thromboxane A2. This has led to the development of drugs such as prostacyclin analogs epoprostenol, iloprost, and treprostinol, which have potent vasodilatory and antiproliferative effects. Some of these drugs are difficult to administer, have several side effects, and are generally expensive.[3]

Sildenafil appears to be an important therapeutic addition for patients with pulmonary arterial hypertension. It has the advantage of having a low incidence of side effects, is easy to administer (oral dosage form), and is much cheaper compared to many of the alternatives. It acts by inhibiting phosphodiesterase-5 and prevents breakdown of cyclic guanosine monophosphate, leading to an increase in the activity of endogeneous nitric oxide, which has a potent pulmonary vasodilatory effect. Sildenafil administered orally has been shown to have beneficial effects in patients with PAH, even during treatment with inhaled NO and inhaled iloprost.[1] Sildenafil also prevents rebound pulmonary vasoconstriction on withdrawal of inhaled NO.[4] It has also been shown to increase the overall survival rate in patients with PAH, and may have some utility as an alternative agent for the patient's refractory to other agents such as epoprostenol and inhaled nitric oxide.[1,4] According to the Cochrane database of systematic reviews, the safety and effectiveness of sildenafil in the treatment of pulmonary hypertension of the newborn (PPHN), in neonates, has not yet been established and its use should be restricted within the context of randomized controlled trials. Furthermore, randomized controlled trials of adequate power are required comparing sildenafil with other pulmonary vasodilators in PPHN in neonates.[5]

Sildenafil is a more potent acute pulmonary vasodilator than inhaled nitric oxide, however, it is not pulmonary vascular specific.[4] The main adverse effects of sildenafil include priapism, angina pectoris, arrhythmias, systemic hypotension, urticaria, and flushing. Others include sweating, tachycardia, and palpitations.[6] Some of these adverse reactions overlap with the signs and symptoms seen in congestive cardiac failure in infants. In our patient it was not clear whether persistent tachycardia and sweating were due to uncontrolled cardiac failure or due to sildenafil. As the general condition of the child had improved and his tachypnea and hepatomegaly had decreased, sildenafil was omitted with a decrease in tachycardia and sweating. Furthermore, re-challenge was not done in the interest of the patient, fearing reappearance of adverse drug reaction and ethical constraints. Thus, the appearance of sweating and tachycardia in a patient taking sildenafil, who does not have other signs of cardiac failure, could not be explained by a concurrent disease, drug or chemicals, when a dechallenge improved the condition. This reaction is dose unrelated and can be labeled as a type B class of adverse effect.[7] It can also be considered as probable or likely, as per causality assessment.[8]

Sildenafil is a drug used uncommonly in children, mostly for PAH associated with congenital heart disease. Hence one can face this diagnostic dilemma in clinical practice and the treating pediatrician should be aware of these adverse reactions of sildenafil. When one faces such a situation, then the general activity of the patient, feeding, and other parameters like tachypnea and hepatomegaly should be assessed. If the child is showing improvement in these parameters then the possibility of an adverse reaction to sildenafil should be kept in mind. This will avoid overtreatment and misdiagnosis in such patients.
